# A Cost-Effective Screening Inflammation Indicator for Atopic Dermatitis Suitable for Primary Care and Self-Assessment

**DOI:** 10.3390/diagnostics15192483

**Published:** 2025-09-28

**Authors:** Chengbin Ye, Xuyang Zhou, Ying Zou

**Affiliations:** Allergic Dermatoses Clinical Center, Shanghai Skin Disease Hospital, Tongji University School of Medicine, Shanghai 200443, China; tinylearner001@163.com (C.Y.); 2231162@tongji.edu.cn (X.Z.)

**Keywords:** atopic dermatitis, cross-sectional study, atopic inflammation index, cost-effective, biomarkers

## Abstract

**Background/Objectives**: Atopic dermatitis (AD), a chronic inflammatory skin condition, significantly impairs quality of life but remains underdiagnosed in primary care. Blood-cell-count-derived inflammatory indices are emerging as cost-effective biomarkers, but their pathological relevance to AD is limited and requires further discussion. **Methods**: We developed the Atopic Inflammation Index (AII), a novel blood-cell-based biomarker reflecting AD pathogenesis, and initially assessed its levels in AD patients and healthy controls using clinical samples from Shanghai, China. We then analyzed data from the NHANES (National Health and Nutrition Examination Survey) 2005–2006 cohort (*n* = 6855) to verify the AII-AD association and compared AII’s diagnostic performance with IgE and eosinophils. **Results**: Clinical analysis showed a nonlinear association between AII and AD severity. AII effectively distinguished AD patients (including mild cases) from healthy controls (*p* < 0.001) without elevation in psoriasis or urticaria, unlike eosinophils. In NHANES 2005–2006 (*n* = 720 AD cases, 10.5%), AII levels were higher in AD compared to non-AD patients (2.33 [1.39–4.09] vs. 2.03 [1.19–3.49], *p* = 0.007) and remained independently associated after adjustment (OR = 1.03, 95%CI = 1.01–1.04, *p* = 0.003), while IgE/eosinophils showed non-significant trends. Restricted cubic splines confirmed linear prediction (*p* = 0.006), and subgroup analyses supported consistency (*P*-interaction > 0.05). AII outperformed eosinophils (AUC:0.568 vs. 0.546, *p* = 0.025) with improved detection (sensitivity 0.361→0.614). Sensitivity analysis confirmed robustness after excluding medications, chronic diseases and adult populations. **Conclusions**: AII is stable and reliable in screening and diagnosing AD, offering a low-cost, practical solution for primary care. This verifies the feasibility of integrating existing detection indicators into new biomarkers, providing valuable inspiration for precision medicine research.

## 1. Introduction

Atopic dermatitis (AD), also known as atopic eczema, is a chronic, recurrent inflammatory skin disease frequently associated with asthma and allergic rhinitis [1]. AD can occur at any age but typically begins in infancy. Until the early 21st century, AD was believed to be more prevalent in developed countries, with a lifetime prevalence of 15–20% [2]. However, the 25th World Congress of Dermatology identified this as a misconception after analyzing Global Burden of Disease (GBD) data and finding substantial indirect evidence. Weak healthcare infrastructure, limited physician availability and inadequate training for diagnosis and treatment in low-income environments have resulted in a significant underestimation of AD’s prevalence [3]. Currently, there is no cure for AD, and its symptoms, including severe itching, significantly impact patients’ quality of life, ranking first among non-lethal disease burdens [4]. Despite increasing attention from dermatologists, a substantial rate of missed cases or misdiagnoses remains, particularly in primary care settings [5]. The main reason for this is that traditional diagnostic criteria, such as those by Hanifin and Rajka and Williams, rely on clinical signs, and professionals in primary care institutions often lack the necessary experience [6]. Additionally, the pathology and laboratory results of AD are not specific. This results in poor disease control and significant socioeconomic burdens. Therefore, there is an urgent need for accurate and convenient screening methods to ensure timely treatment for potential patients.

AD was initially considered an IgE-mediated inflammatory skin disease, leading to the use of serum total IgE for diagnosis and prognosis assessment [7,8]. However, this indicator is not elevated in over 20% of AD patients, indicating a bias in understanding [9]. Non-AD patients with asthma and allergic rhinitis may also exhibit elevated IgE levels. Moreover, it is difficult to access this test in resource-limited settings, such as rural areas in China. Recent research among scholars suggests that AD is a complex disease primarily driven by Th2 inflammation and involving multiple immune pathways [10]. Thus, considerable efforts have been made to identify potential biomarkers related to these immune pathways [11]. Currently, only CCL17 (Chemokine (C-C motif) ligand 17)/TARC (Thymus and activation-regulated chemokine) has been confirmed to correlate closely with disease severity and is included as a diagnostic biomarker in Japanese guidelines [12]. However, due to insufficient clinical validation and high testing costs, the clinical application of these biomarkers will take time. Eosinophils are also an important part of type 2 inflammation. The Chinese AD diagnostic criteria proposed by Zhang et al. consider eosinophils as equally important as IgE in their significant correlation with disease severity [13,14]. Eosinophil counts are easy to obtain and low-cost, but they identify AD less effectively. Improving the diagnostic efficiency of eosinophils could enhance the screening accuracy for AD.

Blood-cell-count-derived biomarkers, such as the Systemic Immune-Inflammation Index (SII) and the Systemic Inflammatory Response Index (SIRI), are cost-effective [15,16]. This is attributed to the incorporation of complete blood counts in routine health check-ups, a procedure that nearly everyone has undergone without requiring supplementary testing. Biomarkers derived from these blood cell counts have been validated across a range of diseases, encompassing both cardiovascular and cerebrovascular conditions; however, their alignment with the underlying mechanisms of AD remains incomplete. The eosinophil-to-lymphocyte ratio (ELR) shows promise in small studies, but its diagnostic capacity compared to traditional biomarkers remains unclear [17,18]. This study aims to explore new biomarkers for AD screening using existing testing indicators, proposing a cost-effective and high-performance index to enhance AD detection rates in primary healthcare and routine examinations with limited resources.

## 2. Materials and Methods

### 2.1. Data Sources

This retrospective study analyzed de-identified data from patients who underwent routine blood tests at Shanghai Skin Disease Hospital (Shanghai, China) between October 2022 and May 2025. Demographic data collected included age and gender. The study was approved by the local ethics committee of Shanghai Skin Disease Hospital (Approval No: 2022-27).

The National Health and Nutrition Examination Survey (NHANES), conducted biennially by the U.S. National Center for Health Statistics (NCHS), is a nationally representative survey employing stratified, multistage probability cluster sampling to assess the health and nutritional status of the non-institutionalized U.S. population. The NCHS Research Ethics Review Board approved the NHANES protocol, and all participants provided written informed consent upon enrollment (Protocol #2005-06). This study adheres to the STROBE (Strengthening the Reporting of Observational Studies in Epidemiology) guidelines for reporting observational epidemiological research.

### 2.2. Study Design and Population

Clinical samples included subjects with complete blood routine reports. The final dataset included 151 controls, 286 AD patients, 152 psoriasis vulgaris patients and 152 chronic urticaria patients. The severity of AD patients was assessed using the Eczema Area and Severity Index (EASI), with EASI ≤ 7 classified as mild and EASI ≥ 8 classified as moderate-to-severe cases. In total, 180 patients were classified as mild AD, and 106 as moderate-to-severe AD.

**Figure 1:**
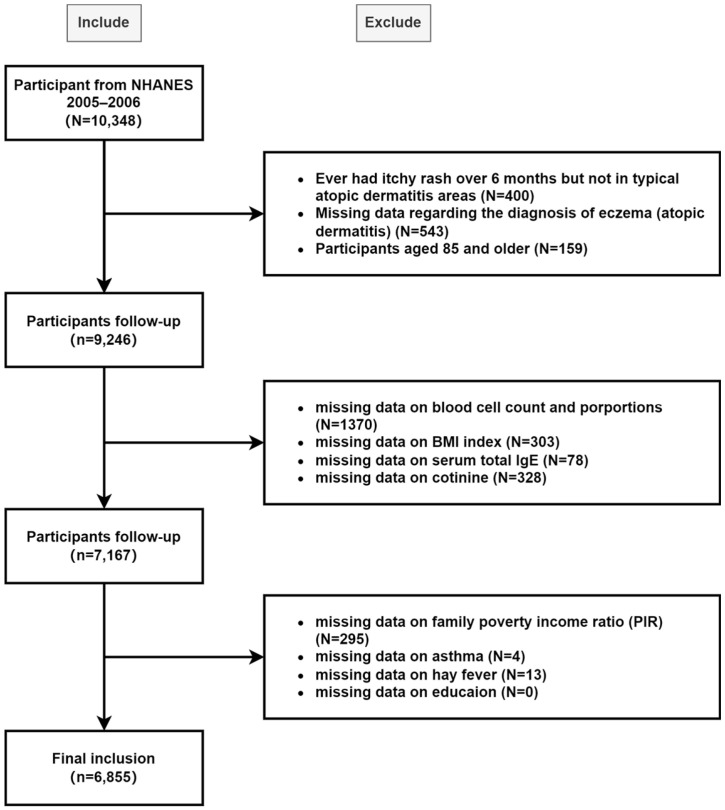
Flow chart of participant selection from the NHANES, 2005–2006.

The NHANES database included 10,348 subjects from the 2005–2006 cycle: the only cycle containing comprehensive dermatology questionnaires. Exclusion criteria were as follows: missing diagnostic information on atopic dermatitis; age ≥ 85 years (all individuals aged 85 or older were recorded as 85 in the dataset regardless of their actual age); insufficient information on complete blood cell counts, total IgE or cotinine (nicotine metabolites served as biomarkers for nicotine dependence and smoking status); or lack of data on Body Mass Index (BMI), family poverty income ratio (PIR), asthma history or educational level. The final study population comprised 6855 individuals. The process of recruitment of NHANES is shown in Figure 1.

### 2.3. Definition of Atopic Dermatitis

All AD cases from hospital were diagnosed by outpatient dermatologists based on the criteria of Williams et al. [19]. AD in the 2005–2006 NHANES was assessed via self-report questionnaires and diagnosed based on affirmative responses to question one, or positive answers to both questions two and three: (1) “Has a doctor or other health professional ever told {you/SP} that {you have/SP s/he has} eczema?”; (2) “Have you/Has SP ever had an itchy rash lasting at least 6 months?”; and (3) “Has this itchy rash ever affected any of the following areas: elbow folds, behind the knees, front of the ankles, under the buttocks, or around the neck, ears, or eyes?” [20]. This definition is considered to meet the Hanifin and Rajka criteria and the Williams criteria, which is analogous to having three of the Hanifin and Rajka major criteria: chronic dermatitis, flexural involvement and pruritus [21].

### 2.4. Exposure Variable

Blood cell counts were measured using automated hematology analyzing devices. As the NHANES database provides only one decimal place for cell counts, which fails to effectively reflect blood count differences, we recalculated the results using cell proportions and retained two decimal places. The following formulas were used to calculate immune-inflammatory markers: (1) Systemic Immune-Inflammation Index: $SII=\frac{neutrophil\times platelet}{lymphocyte}$; (2) Systemic Inflammation Response Index: $SIRI=\frac{neutrophil\times monocyte}{lymphocyte}$; (3) Systemic Inflammatory Composite Index: $AISI=\frac{neutrophil\times monocyte\times platelet}{lymphocyte}$; (4) Other inflammatory indices based on cell count ratios include $NLR=\frac{neutrophil}{lymphocyte}$, $PLR=\frac{platelet}{lymphocyte}$, $PMR=\frac{platelet}{monocyte}$, $MLR=\frac{monocyte}{lymphocyte}$, $LMR=\frac{lymphocyte}{monocyte}$, $BLR=\frac{basophil}{lymphocyte}$, $ELR=\frac{eosinophil}{lymphocyte}$.

Based on the pathogenesis of atopic dermatitis, we designed new biomarkers that integrate the assessment of cells involved in type 2 inflammation, while also adjusting for the inflammatory background (through SIRI) and nutritional status (through mean corpuscular hemoglobin, MCH). This biomarker termed the Atopic Inflammation Index (AII) is calculated as follows: $AII=\frac{\left(eosinophil+basophil\right)\times platelet}{SIRI\times MCH}$.

### 2.5. Covariates

In clinical samples, variables included gender, age and atopic dermatitis (AD) severity scores, such as EASI and SCORAD (Scoring Atopic Dermatitis). Based on the literature, the covariates in NHANES included were age, gender, race, education level, family income, BMI, smoking status and asthma history [22]. According to atopic dermatitis guidelines, this study categorized age into four groups: children (<12), teenagers (≥12 to <18), adults (≥18 to <60), and the elderly (≥60). As used in the NHANES, we categorized the participants into the following five races and ethnicities: Mexican American, non-Hispanic Black, non-Hispanic White, other Hispanic, and other race (including multiracial). Educational level was divided into three levels (less than high school, high school graduate, and college graduate or above). As used by US government departments to report NHANES dietary and health data, we categorized family income into the following three levels based on the family poverty income ratio: low income (<1.3), medium income (≥1.3 to 3.5) and high income (≥3.5). BMI was categorized into the following four groups: underweight (<18.5), healthy weight (≥18.5 to <25), overweight (≥25 to <30) and obese (≥30). Smoking status was classified into four groups based on serum cotinine (nicotine metabolites) levels: non-smoker (with cotinine below the detection limit of 0.011), passive smoker (0.011 < cotinine < 0.1), occasional active smoker (0.1 ≤ cotinine < 30) and nicotine addict (cotinine ≥ 30).

### 2.6. Statistical Methods

Non-normally distributed continuous variables were reported as medians with percentiles and analyzed using the Mann–Whitney test. Categorical variables were analyzed using chi-square or Fisher’s exact tests. Due to limited covariate data, multivariate regression was used to adjust for gender and age in the clinical samples. Differences in AII and eosinophils were compared between patients with varying severities of AD, psoriasis vulgaris, chronic urticaria and healthy controls to assess their performance independently in screening for AD. A restricted cubic spline (RCS) was used to evaluate the impact of AII variation on eosinophil levels, total IgE and disease severity in the atopic dermatitis population.

For the NHANES database, we used the “survey” package in R for complex survey design, weighted data handling and statistical testing. Multivariate logistic regression models were used to assess the association between AII, eosinophil counts, IgE, and AD risk, adjusting for all confounders. Confounding factors such as age, PIR, BMI and cotinine were treated as continuous to minimize information loss and avoid dilution of statistical power. RCS analysis explored nonlinear relationships among AD risk and AII based on the Akaike Information Criterion (AIC), while validating the nonlinear association between AII and traditional biomarkers. Subgroup and interaction analyses enabled the evaluation of associations across demographics. Receiver operator characteristic (ROC) curves assess the specificity and sensitivity of AII in predicting AD risk, comparing it with eosinophil counts and IgE levels. Sensitivity analyses excluded participants using systemic medications in the past month, those with a history of tumors and chronic diseases, and adult populations. Statistical analyses used odds ratios (ORs) and 95% confidence intervals (95%CIs). Two-sided corrected *p* values less than or equal to 0.05 were also considered statistically significant in the hypothesis tests. Data processing and analysis were performed using R version 4.4.3 (28 February 2025), and Zstats 1.0 (www.zstats.net (accessed on 17 March 2025)).

## 3. Results

### 3.1. The Stability and Reliability of AII in Clinical Practice

The clinical samples included 151 healthy controls, 180 mild AD cases, 106 moderate-to-severe AD cases and 152 cases each of psoriasis vulgaris and chronic urticaria. Compared to the healthy control group, significant differences in gender and age were observed across the skin disease groups (all *p* < 0.001) (Table S1). After adjusting for gender and age, AII showed significant differences between mild AD (OR = 1.62, 95% CI = 1.38–1.90, *p* < 0.001) and moderate-to-severe AD groups (OR = 1.90, 95% CI = 1.54–2.34, *p* < 0.001) compared to healthy controls. AII did not exhibit abnormal elevation in psoriasis or chronic urticaria, and the narrow 95% confidence intervals confirmed the robustness and independence of this indicator (Table 1). In contrast, eosinophils were significantly elevated in both AD and non-AD skin disease subgroups compared to healthy controls.

**Figure 2:**
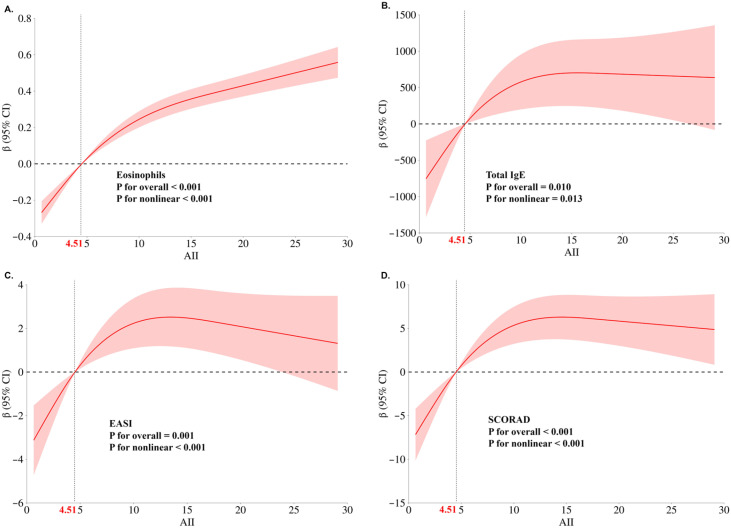
Nonlinear associations between AII and key biomarkers/clinical scores in atopic dermatitis patients. (**A**) The relationship between AII and eosinophils. (**B**) The relationship between AII and total IgE. (**C**) The relationship between AII and EASI (eczema area and severity index). (**D**) The relationship between AII and SCORAD (scoring of atopic dermatitis). Multivariable adjustment was performed for gender and age. The median of AII is considered a reference value and the number of nodes is three.

Focusing on patients with atopic dermatitis, restricted cubic spline analysis assessed the correlation between AII and eosinophils, total IgE, the EASI score and SCORAD score (Figure 2). The results indicated a significant nonlinear relationship between AII and both traditional biomarkers and severity scores. When AII exceeded the median, levels of traditional biomarkers and disease severity increased. However, after AII surpassed approximately 13, changes in disease severity and total IgE became less pronounced.

### 3.2. The Independent Predictive Role of AII in AD Patients

To further clarify the relationship between AII and AD with a larger sample size and more covariates, a cohort of 6855 individuals was selected from the 2005–2006 NHANES, including 720 AD patients and 6135 non-AD individuals. The results indicate that among the U.S. population, AD is more prevalent in children, non-Hispanic whites, individuals with a low BMI and those with a history of asthma or hay-fever (Table 2).

Blood tests showed that AII was significantly higher in AD patients (2.33 [1.39–4.09] vs. non-AD patients: 2.03 [1.19–3.49], *p* = 0.007), while eosinophil and other inflammatory indices showed no differences except for the neutrophil-to-lymphocyte ratio (NLR) (Table 2 and Table S2). Three regression models assessed AII’s, IgE’s and eosinophils’ association with AD risk (Table 3). After adjusting for all confounders, AII remained significant (OR = 1.03, 95%CI = 1.01–1.04, *p* = 0.003), while IgE and eosinophils lost significance after adjusting for allergy history. AII’s OR and 95% CI showed consistent trends across models.

### 3.3. The Linear Relationship Between AII and AD Risk and Subgroup Analysis

**Figure 3:**
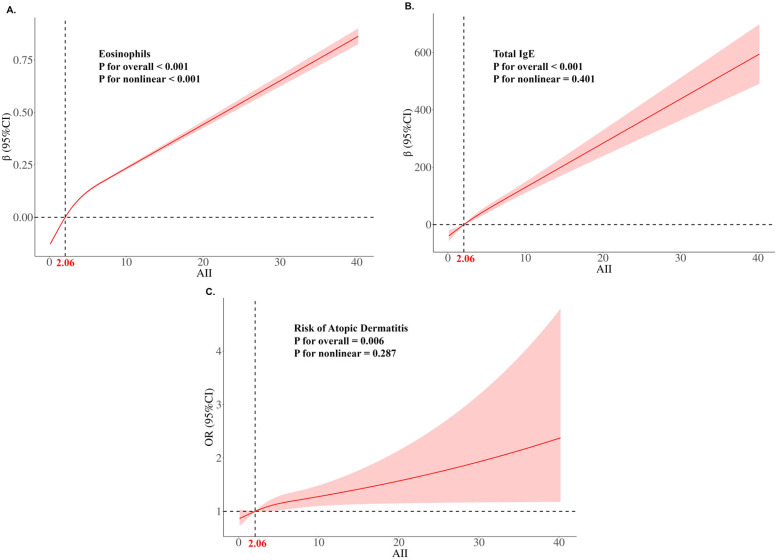
Restricted cubic spline regression on the relationship between AII and biomarkers or disease risk of atopic dermatitis in the U.S. population. (**A**) The relationship between AII and eosinophils. (**B**) The relationship between AII and total IgE. (**C**) The relationship between AII and risk of atopic dermatitis. Multivariable adjustment was performed for gender, race, age, education, Body Mass Index (BMI), family poverty income ratio (PIR), cotinine, asthma and hay-fever. The median of AII is 2.06 and is considered a reference value. According to the Akaike Information Criterion (AIC), the optimal number of nodes of the RCS curve is determined to be three.

The RCS analysis conducted within the U.S. population revealed a significant association between AII and traditional biomarkers, as well as the risk of AD (Figure 3). Remarkably, both total IgE levels and the risk of AD exhibited a linear relationship with AII. This finding implies that as AII increases, there is a corresponding rise in both the likelihood of developing AD and the levels of total IgE.

**Figure 4:**
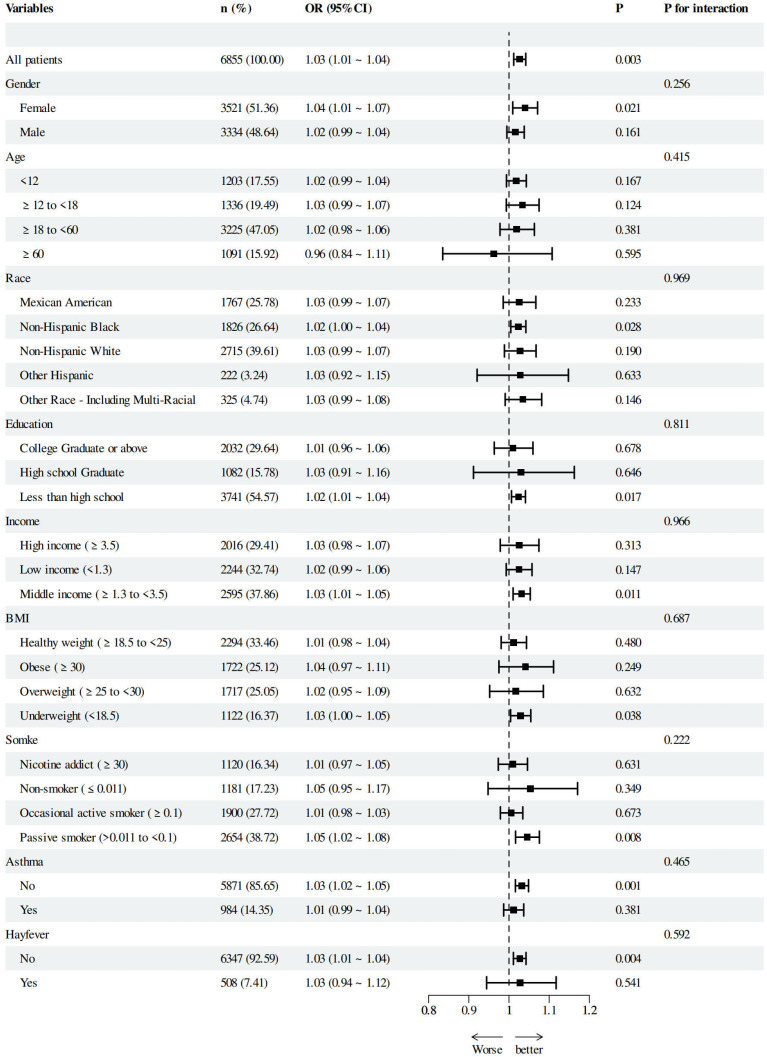
Forest plot for subgroup analysis of associations between AII and atopic dermatitis. Income was categorized based on the family poverty income ratio (PIR), and smoking status was grouped according to serum cotinine (nicotine metabolites). Odds ratios (ORs) were calculated using multivariate logistics models adjusted for variables in Model 3, except for the variable used for stratification.

Subgroup analysis assessed AII’s association with AD risk across age, gender, race, education, income, BMI, smoking and history of asthma and hay-fever (Figure 4). Elevated AII was an independent risk factor for AD in female, non-Hispanic Blacks, those with less than high school education, households with moderate income, individuals with a low BMI, passive smokers and patients without a history of asthma or hay-fever. Interaction analysis revealed no significant interactions, confirming AII’s independent contribution to assessing AD risk.

### 3.4. The Efficacy of AII in AD Screening and Sensitivity Analysis

**Figure 5:**
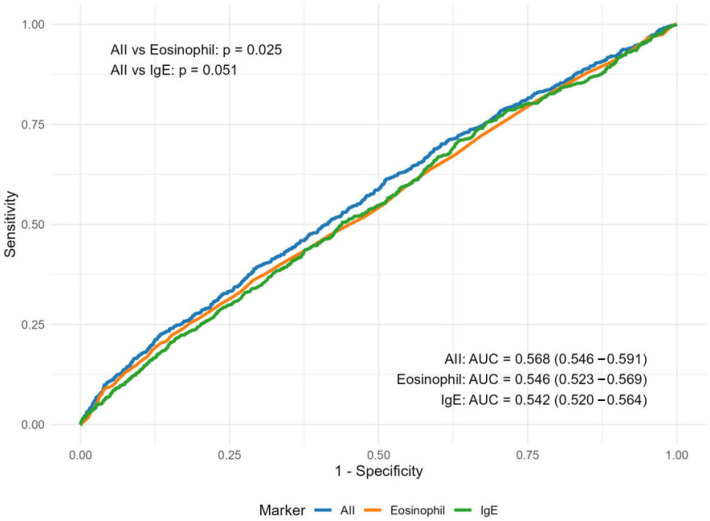
ROC curves and AUC comparison of AII, eosinophil and IgE. Multivariate adjustments were made after weighting for gender, race, age, education, PIR, BMI, cotinine, asthma and hay-fever.

ROC curve analysis demonstrated that AII’s performance is not significantly different from IgE and is superior to eosinophils (AUC: 0.568 vs. 0.546, *p* = 0.025) (Figure 5). In early AD screening, AII demonstrated superior sensitivity compared to eosinophils (0.614 vs. 0.361) (Table S3). In sensitivity analysis, we excluded individuals using systemic medications in the past month, and a significant positive correlation was found between AII and AD risk (OR = 1.03, 95%CI = 1.01–1.05, *p* = 0.002) (Table S4). Furthermore, the sensitivity analyses reaffirmed the robustness of AII, demonstrating that it remained unaffected by a history of chronic disease and tumors (OR = 1.03, 95%CI = 1.01–1.05, *p* = 0.005). Despite reduced precision due to a smaller sample size, the association of AII in the pediatric population was consistent with the overall trend (OR = 1.02, 95% CI = 1.01–1.05, *p* = 0.040).

## 4. Discussion

This study utilized clinical samples and the NHANES database to develop and validate AII as a marker to distinguish AD patients from non-AD individuals. AII demonstrated comparable stability and diagnostic accuracy to IgE, outperforming eosinophils. By expanding the applicability of blood routine tests in the diagnosis of AD, AII offers the advantages of rapid accessibility and low cost, making it suitable for primary care screening and health examinations.

### 4.1. AII Reflects AD-Specific Type 2 Inflammation Through the Evaluation of AD-Related Cells

In the NHANES analysis, most inflammatory markers did not demonstrate significant utility in distinguishing AD from healthy controls. This differs from previous NHANES study results, as their diagnostic criteria for AD have been overly lenient, exaggerating the correlation between SII and AD [23]. Another reason for this lack of significance is that most existing inflammation indices were initially designed to assess chronic inflammation related to cardiovascular diseases and cancer, focusing on cell populations that are not central to the pathogenesis of AD, such as neutrophils. Cells highly associated with AD are mostly overlooked, such as eosinophils.

Compared to existing peripheral blood-cell-count-derived inflammatory indicators such as SII and SIRI, AII aligns more closely with the pathophysiological mechanisms of AD. Eosinophils and basophils are the primary markers of type 2 inflammation, playing synergistic roles in AD pathogenesis, particularly in inducing acute and chronic pruritus [24]. The combined effects of these two cell counts reflect high-risk signals related to allergy and type 2 inflammation [25]. Platelets, which are closely associated with severe allergic conditions, may contribute to type 2 inflammation by releasing inflammatory mediators such as chemokines and cytokines [26]. A comprehensive assessment of these type 2 inflammatory cells is more reliable than evaluating them in isolation, though it is necessary to exclude the interference of non-specific inflammation.

SIRI, which is commonly used to evaluate systemic inflammation, did not show elevated levels in AD patients, suggesting that it primarily reflects non-specific inflammation rather than immune-specific responses. Although SIRI cannot distinguish immune related inflammation (e.g., type 1 vs. type 2), Qi et al. demonstrated a good correlation between SIRI and cytokines/inflammatory factors such as CCL17, CCL22, CCL18 and IL-10 [27]. MCH, which remains relatively stable across different ages and genders compared to hemoglobin levels, is associated with nutritional status and chronic inflammation. We incorporated cells involved in type 2 inflammation while adjusting for systemic inflammation (via SIRI) and nutritional status (via MCH), partially reflecting the complex immune levels of AD. At present, new inflammatory indicators comprehensively assess inflammation and nutritional status to reflect the complexity of the disease, such as the C-reactive protein–albumin–lymphocyte index (CALLY) and the hemoglobin–albumin–lymphocyte–platelet index (HALP) [28,29].

### 4.2. AII Detected Mild AD Cases and Did Not Overlap with Other Inflammatory Skin Diseases

It is worth noting that in clinical practice, AD patients are mainly diagnosed with mild-to-moderate, especially in primary medical institutions. Current small-sample clinical studies related to inflammatory markers derived from blood cells focus on patients with moderate to severe AD, neglecting the much larger group of patients with mild symptoms who require early intervention [17]. The difficulty in conducting AD screening lies in the fact that subtle allergic reactions of patients with mild symptoms can easily be masked by other pathological conditions. This explains why inflammatory indicators such as NLR in the non-AD group were higher than those in the AD group. AII’s ability to correct for non-specific inflammation and nutritional status demonstrates good stability and excellent discriminative ability. In clinical samples, AII levels in mild AD are comparable to those found in moderate to severe cases, ensuring high detectability.

Our clinical data indicate that AII levels are elevated in AD patients but remain within normal ranges in psoriasis, chronic urticaria and healthy subjects. This finding could have the potential to enhance the disease specificity of AII, suggesting its potential for broad application in skin disease screening. While unmeasured confounders cannot be ruled out, the consistency of AII’s associations across AD supports its independent utility. We acknowledge that the high OR value of eosinophils is primarily due to the near absence of high-level samples in the healthy control group. It is well-established that IgE and eosinophil levels may be significantly elevated in other dermatological conditions, particularly in chronic urticaria, which complicates differential diagnosis using traditional indicators. AII could serve to address this diagnostic shortcoming. Although the limited variety of dermatological conditions represented in the NHANES database restricts broader exploration, our findings indicate that AII maintains a statistically significant difference and a narrow confidence interval when distinguishing between AD and healthy individuals, indicating the stability and reliability of these outcomes.

### 4.3. AII Correlates with Disease Severity, Independently Predicts AD and Outperforms Traditional Biomarkers

Our study found that changes in AII among AD patients are significantly correlated with traditional indicators and disease severity trends. In clinical samples, as AII increases, both IgE levels and severity scores rise, and risk prediction stabilizes after reaching a threshold. This threshold may indicate extreme severity in AD patients, suggesting that AII may not fully capture the complexity of their immune status. Although this finding is limited by sample size and requires further validation, this threshold holds potential for precise stratification of AD and guiding stepwise treatment choices for primary care physicians. The NHANES data lacks clinical severity scores, yet the correlation between AII and IgE aligns with expectations. However, due to the absence of severe patients in the NHANES, changes in AII’s risk prediction did not exhibit a plateau. Further prospective multicenter cohort studies are needed to clarify the relationship between AII and disease severity. This could enhance accurate diagnosis in AD screening, aid in disease stratification and treatment guidance and predict therapeutic outcomes.

The relationship between AII and the risk of AD in the NHANES data is both linear and stable, remaining unaffected by demographic factors, smoking history, allergy history, medication use, and tumors or chronic conditions. In contrast, eosinophils and IgE levels lost statistical significance after adjusting for covariates in the NHANES sample. It is evident that in non-AD patients who also suffer from concurrent allergic conditions such as allergic rhinitis or asthma, elevated eosinophil and IgE levels may lead to potential misinterpretations. Within this framework, AII has a higher likelihood of avoiding such errors, providing clinicians with a reliable diagnostic basis. Therefore, while AII’s diagnostic efficacy significantly surpasses that of eosinophils, it does not exceed that of IgE, and still possesses ample clinical utility.

### 4.4. Strengths, Limitations and Future Directions

Reducing the cost of implementating precision medicine while maintaining stability is an advantage of AII. The introduction of AII as a powerful tool for primary care screening is expected to enhance grassroots physicians’ focus on AD, facilitating early intervention. Previous exploration of precision medicine has been dedicated to using multi-omics techniques to explore more microscopic molecules, gene phenotypes, etc., as biomarkers [30]. Popular inflammatory biomarkers include Th2 inflammation markers and the filaggrin gene [31]. While cytokines and other candidate biomarkers provide mechanistic insights, their clinical application is limited by cost and a lack of standardization, making their adoption in primary healthcare settings unrealistic. In contrast, blood-cell-count-derived markers are inexpensive, reproducible, stable and easy to implement, significantly reducing healthcare costs for patients and society. For institutions that cannot perform IgE testing, AII provides an efficient and cost-effective means of screening for AD. For institutions that can conduct IgE testing, using AII in conjunction with IgE can enhance diagnostic efficacy. For patients, it is possible to make a preliminary assessment of AD risk based on previous complete blood count reports to determine whether further testing and evaluation are needed.

Our study has limitations. First, the clinical samples were only adjusted for basic variables, which may introduce potential biases in the results. Second, AD diagnosis in NHANES relied on self-reports, thereby introducing recall bias. Third, the moderate predictive value of AII requires further validation in large-scale clinical samples. Fourth, specific subgroups of atopic dermatitis, such as atopic chronic hand eczema, were not assessed, limiting the study’s applicability [32]. Finally, the cross-sectional design of NHANES precludes establishing causality or tracking AD severity changes.

Enhancing predictive accuracy and broadening application scenarios are key future directions for low-cost biomarkers. Both domestic and international teams are exploring high-cost biomarkers, such as serum cytokines, for precise classification and efficacy prediction in AD [33,34]. However, studies show that inflammatory indicators from blood cells, like ELR, can also predict the efficacy of targeted therapies and small molecule drugs [17,18]. This suggests that AII can serve as an effective complement to biomarkers like cytokines and genetic phenotypes, facilitating precise disease stratification and identifying the most beneficial targeted therapies for specific patients. Future research will include multicenter prospective cohort studies to evaluate AII’s predictive capabilities and efficacy detection. We will also utilize machine learning with clinical variables to enhance AII’s accuracy, integrate existing hematological indicators to refine AII, and develop more cost-effective biomarkers. We hope our preliminary findings will provide valuable insights for the advancement of precision diagnosis and treatment, ultimately helping to alleviate socioeconomic burdens.

## 5. Conclusions

The AII demonstrates superior diagnostic accuracy and robustness for AD screening compared to traditional biomarkers like eosinophils, thereby enhancing the clinical utility of complete blood counts. Its alignment with AD pathophysiology, along with cost-effectiveness and practicality, makes it a promising tool for primary care and routine health check-ups. As a low-cost complement to currently popular biomarkers like cytokines and gene phenotypes, the design concept of this inflammatory indicator is expected to provide reference for the exploration of predictive factors in precision medicine.

## Figures and Tables

**Table 1:** Statistical comparison of eosinophil and AII among different skin disease groups in patients.

Variables	Total	Eosinophil			AII		
		Value, M (Q_1_, Q_3_)	OR (95%CI) ^a^	*p*	Value, M (Q_1_, Q_3_)	OR (95%CI) ^a^	*p*
Healthy controls	151	0.10 (0.06, 0.15)	1.00 (Reference)		1.66 (0.97, 2.64)	1.00 (Reference)	
Mild AD	180	0.27 (0.14, 0.47)	37,828.73 (2069.22~691,571.70)	<0.001 *	4.41 (1.81, 7.97)	1.62 (1.38~1.90)	<0.001 *
Moderate to severe AD	106	0.48 (0.31, 0.66)	1,243,099.66 (28,801.34~53,653,648.47)	<0.001 *	4.77 (2.48, 9.39)	1.90 (1.54~2.34)	<0.001 *
Psoriasis vulgaris	152	0.15 (0.08, 0.24)	84.98 (3.38~2134.56)	0.007 *	1.54 (0.98, 2.83)	1.18 (0.99~1.40)	0.061
Chronic urticaria	152	0.12 (0.08, 0.22)	47.78 (4.37~522.10)	0.002 *	1.45 (0.83, 2.56)	1.03 (0.90~1.18)	0.639

M: Median, Q_1_: 1st Quartile, Q_3_: 3rd Quartile, OR: odds ratio, CI: confidence interval, *: *p* < 0.05; AII, Atopic Inflammatory Index; AD, atopic dermatitis; ^a^, Multivariable logistic regression models were constructed and adjusted for age and gender.

**Table 2:** Characteristics of participants and blood test results in the NHANES 2005–2006 cycles.

Variable	Total (*n* = 6855)	Non-Atopic Dermatitis (*n* = 6135)	Atopic Dermatitis (*n* = 720)	Statistic	*p*
Age, M (Q_1_, Q_3_)	38.00 (21.00, 53.00)	38.00 (21.00, 53.00)	36.00 (15.00, 53.00)	Z = −2.06	0.040 *
Age, *n* (%)				χ^2^ = 46.61	<0.001 *
<12	1203 (11.05)	1014 (10.23)	189 (18.05)		
≥12 to <18	1336 (9.23)	1178 (9.01)	158 (11.15)		
≥18 to <60	3225 (63.64)	2943 (64.47)	282 (56.50)		
≥60	1091 (16.08)	1000 (16.29)	91 (14.30)		
Gender, *n* (%)				χ^2^ = 2.53	0.151
Female	3521 (51.38)	3138 (51.05)	383 (54.19)		
Male	3334 (48.62)	2997 (48.95)	337 (45.81)		
Race, *n* (%)				χ^2^ = 19.31	0.002 *
Mexican American	1767 (9.07)	1662 (9.52)	105 (5.22)		
Non-Hispanic Black	1826 (11.83)	1583 (11.72)	243 (12.83)		
Non-Hispanic White	2715 (69.89)	2396 (69.34)	319 (74.59)		
Other Hispanic	222 (3.63)	200 (3.73)	22 (2.72)		
Other Race—Including Multi-Racial	325 (5.58)	294 (5.69)	31 (4.65)		
Education, *n* (%)				χ^2^ = 17.33	0.094
College graduate or above	2032 (45.85)	1830 (46.16)	202 (43.14)		
High school graduate	1082 (19.98)	1003 (20.43)	79 (16.12)		
Less than high school	3741 (34.17)	3302 (33.41)	439 (40.74)		
Family income, *n* (%)				χ^2^ = 3.67	0.314
Low	2244 (19.05)	2020 (19.03)	224 (19.24)		
Medium	2595 (37.62)	2342 (37.98)	253 (34.50)		
High	2016 (43.33)	1773 (42.99)	243 (46.27)		
Body Mass Index (BMI), *n* (%)				χ^2^ = 27.27	<0.001 *
Underweight	1122 (11.07)	955 (10.47)	167 (16.25)		
Healthy weight	2294 (32.74)	2057 (32.53)	237 (34.52)		
Overweight	1717 (27.65)	1557 (28.13)	160 (23.49)		
Obese	1722 (28.54)	1566 (28.87)	156 (25.75)		
Smoke, *n* (%)				χ^2^ = 4.57	0.291
Non-smoker	1181 (17.10)	1063 (16.84)	118 (19.31)		
Passive smoker	2654 (38.24)	2367 (38.13)	287 (39.23)		
Occasional active smoker	1900 (22.44)	1691 (22.56)	209 (21.41)		
Nicotine addict	1120 (22.22)	1014 (22.47)	106 (20.05)		
Asthma, *n* (%)				χ^2^ = 33.89	<0.001 *
No	5871 (85.67)	5326 (86.51)	545 (78.45)		
Yes	984 (14.33)	809 (13.49)	175 (21.55)		
Hay-fever, *n* (%)				χ^2^ = 32.10	<0.001 *
No	6347 (89.07)	5716 (89.80)	631 (82.82)		
Yes	508 (10.93)	419 (10.20)	89 (17.18)		
Biomarker, *n* (%)					
IgE	39.40 (14.80, 111.00)	39.00 (14.60, 110.00)	43.90 (17.20, 124.00)	Z = −2.40	0.016 *
Eosinophil	0.17 (0.11, 0.27)	0.17 (0.11, 0.27)	0.18 (0.12, 0.28)	Z = −1.54	0.125
AII	2.06 (1.22, 3.56)	2.03 (1.19, 3.49)	2.33 (1.39, 4.09)	Z = −2.70	0.007 *

M: Median, Q_1_: 1st Quartile, Q_3_: 3rd Quartile, Z: Mann–Whitney test, χ^2^: Chi-square test, *: *p* < 0.05.

**Table 3:** Univariate and multivariate analyses using the activity-stratified weighted logistic regression model.

Variables ^a^	Model1 ^b^		Model2 ^c^		Model3 ^d^	
	OR (95%CI)	*p*	OR (95%CI)	*p*	OR (95%CI)	*p*
AII						
Continuous	1.04 (1.02~1.05)	<0.001 *	1.03 (1.02~1.05)	<0.001 *	1.03 (1.01~1.04)	0.003 *
Q1	1.00 (Reference)		1.00 (Reference)		1.00 (Reference)	
Q2	1.10 (0.73~1.65)	0.646	1.09 (0.71~1.66)	0.697	1.07 (0.71~1.63)	0.740
Q3	1.26 (0.94~1.67)	0.138	1.24 (0.90~1.69)	0.204	1.16 (0.85~1.57)	0.357
Q4	1.59 (1.21~2.08)	0.004 *	1.47 (1.04~2.08)	0.044 *	1.37 (0.99~1.90)	0.075
P for trend		0.006 *		0.043 *		0.081
IgE						
Continuous	1.01 (1.01~1.01)	0.025 *	1.01 (1.01~1.01)	0.019 *	1.00 (1.00~1.00)	0.055
Q1	1.00 (Reference)		1.00 (Reference)		1.00 (Reference)	
Q2	1.18 (0.89~1.55)	0.266	1.24 (0.94~1.62)	0.146	1.22 (0.92~1.61)	0.187
Q3	1.22 (0.97~1.52)	0.108	1.33 (1.05~1.69)	0.030 *	1.24 (0.97~1.60)	0.113
Q4	1.27 (1.09~1.49)	0.009 *	1.43 (1.22~1.68)	<0.001 *	1.24 (1.05~1.46)	0.022 *
P for trend		0.073		0.009 *		0.192
Eosinophil						
Continuous	1.96 (1.19~3.23)	0.018 *	2.02 (1.23~3.30)	0.014 *	1.62 (0.98~2.68)	0.081
Q1	1.00 (Reference)		1.00 (Reference)		1.00 (Reference)	
Q2	1.15 (0.86~1.53)	0.354	1.18 (0.88~1.59)	0.281	1.16 (0.87~1.55)	0.324
Q3	1.00 (0.73~1.36)	0.983	1.05 (0.76~1.45)	0.790	0.98 (0.71~1.34)	0.891
Q4	1.26 (0.93~1.71)	0.163	1.31 (0.94~1.82)	0.130	1.17 (0.84~1.62)	0.373
P for trend		0.316		0.269		0.620

OR: odds ratio, CI: confidence interval, *: *p* < 0.05. ^a^ Evaluation of continuous variables and the trend of *p*-values based on quartiles and medians. ^b^ Crude model. ^c^ Adjust for gender, race, age, education, PIR, BMI and cotinine. ^d^ Adjust for gender, race, age, education, PIR, BMI, cotinine, asthma and hay-fever.

## Data Availability

The data that support the findings of this study are available from the corresponding author upon reasonable request.

## Supplementary Materials

The following supporting information can be downloaded at: https://www.mdpi.com/article/10.3390/diagnostics15192483/s1. Table S1: Statistical comparison of demographic characteristics among different skin disease groups in clinical patients. Table S2: Other Inflammatory markers derived from blood cell counts in the NHANES 2005–2006 cycles. Table S3: ROC analysis results for AII, eosinophils, and IgE. Table S4: Association between AII and atopic dermatitis, excluded systemic medication history, chronic diseases, tumor history and adult populations respectively.

## Abbreviations

The following abbreviations are used in this manuscript:

NHANES	National Health and Nutrition Examination Survey
NCHS	National Center for Health Statistics
AD	Atopic Dermatitis
AII	Atopic Inflammation Index
PIR	Poverty Income Ratio
BMI	Body Mass Index
SII	Systemic Immune-Inflammation Index
SIRI	Systemic Inflammation Response Index
AISI	Aggregate Index of Systemic Inflammation
NLR	Neutrophil-to-Lymphocyte Ratio
PLR	Platelet-to-Lymphocyte Ratio
PMR	Platelet-to-Monocyte Ratio
MLR	Monocyte-to-Lymphocyte Ratio
LMR	Lymphocyte-to-Monocyte Ratio
ELR	Eosinophil-to-Lymphocyte Ratio
ENR	Eosinophil-to-Neutrophil Ratio
MCH	Mean Corpuscular Hemoglobin
IgE	Immunoglobulin E
OR	Odds Ratios
CI	Confidence Intervals
RCS	Restricted Cubic Spline
AIC	Akaike Information Criterion
ROC	Receiver Operating Characteristic
AUC	Area Under the Curve
NSAIDs	Non-Steroidal Anti-Inflammatory Drugs
CALLY	C-Reactive Protein–Albumin–Lymphocyte Index
HALP	Hemoglobin–Albumin–Lymphocyte–Platelet Index
