# Supplementary material for: A Cost-Effective Screening Inflammation Indicator for Atopic Dermatitis Suitable for Primary Care and Self-Assessment
Source: Diagnostics (Basel). 2025 Sep 28;15(19):2483. doi: 10.3390/diagnostics15192483 (PMC12523488; doi:10.3390/diagnostics15192483)
Supplement: Supplementary file 1 [file diagnostics-15-02483-s001.zip › diagnostics-3840465-supplementary.pdf]

Table S1. Statistical comparison of demographic characteristics among different skin disease groups in clinical patients.

Variables[a]	Total	Age			Gender			
		Value, M (Q_1, Q_3)	Statistic	P	Female, n(%)	Male, n(%)	Statistic	P
**Healthy controls**	151	30.00 (27.00, 36.00)	ref		126 (83.44)	25 (16.56)	ref	
**Mild AD**	180	18.00 (11.00, 33.00)	Z=-6.99	<.001*	99 (55.00)	81 (45.00)	χ^2=30.52	<.001*
**Moderate to severe AD**	106	24.00 (15.00, 40.75)	Z=-3.62	<.001*	40 (37.74)	66 (62.26)	χ^2=56.89	<.001*
**Psoriasis vulgaris**	152	47.50 (36.50, 62.25)	Z=-9.74	<.001*	50 (32.89)	102 (67.11)	χ^2=79.50	<.001*
**Chronic Urticaria**	152	38.50 (28.00, 54.25)	Z=-4.68	<.001*	102 (67.11)	50 (32.89)	χ^2=10.86	<.001*

M: Median, Q_1: 1st Quartile, Q_3: 3st Quartile, Z: Mann-Whitney test, χ^2: Chi-square test, *: P<0.05; a, All subgroups of skin diseases were compared pairwise with healthy individuals.

Table S2. Other Inflammatory markers derived from blood cell counts in the NHANES 2005–2006 cycles.

Variable[a]	Total (n = 6855)	Participants without dermatoses (n=6135)	Atopic dermatitis (n=720)	Statistic	P
SIRI	1.00 (0.69, 1.44)	1.01 (0.70, 1.44)	0.94 (0.67, 1.43)	Z=-1.63	0.102
AISI	279.82 (185.79, 426.29)	280.98 (186.62, 425.11)	272.72 (176.23, 438.96)	Z=-0.47	0.637
SII	511.62 (368.37, 728.91)	513.13 (368.62, 730.09)	504.00 (358.99, 706.57)	Z=-0.93	0.352
NLR	1.86 (1.37, 2.48)	1.88 (1.38, 2.49)	1.81 (1.28, 2.38)	Z=-1.98	0.047*
PLR	129.64 (104.31, 163.19)	129.70 (104.55, 162.96)	129.13 (101.78, 164.13)	Z=-0.28	0.780
PMR	515.38 (406.06, 661.02)	515.09 (404.69, 658.33)	523.73 (415.12, 693.75)	Z=-0.99	0.324
MLR	0.25 (0.20, 0.32)	0.25 (0.20, 0.32)	0.24 (0.20, 0.32)	Z=-1.09	0.275
LMR	4.00 (3.11, 5.00)	4.00 (3.11, 5.00)	4.14 (3.12, 5.11)	Z=-1.09	0.276
ELR	0.08 (0.05, 0.12)	0.08 (0.05, 0.12)	0.08 (0.05, 0.13)	Z=-1.18	0.238
ENR	0.04 (0.03, 0.07)	0.04 (0.03, 0.07)	0.05 (0.03, 0.08)	Z=-1.92	0.055

M: Median, Q_1: 1st Quartile, Q_3: 3st Quartile, Z: Mann-Whitney test, χ^2: Chi-square test, *: P<0.05

Abbreviations: NHANES, National Health and Nutrition Examination Survey; SII, Systemic Immune-Inflammation Index; SIRI, Systemic Inflammation Response Index; AISI, Aggregate Index of Systemic Inflammation; NLR, Neutrophil-to-Lymphocyte Ratio; PLR, Platelet-to-Lymphocyte Ratio; PMR, Platelet-to-Monocyte Ratio; MLR, Monocyte-to-Lymphocyte Ratio; LMR, Lymphocyte-to-Monocyte Ratio; ELR, Eosinophil-to-Lymphocyte Ratio; ENR, Eosinophil-to-Neutrophil Ratio;

a Blood cell counts were measured using automated hematology analyzing devices. Calculate the actual count based on the percentage of granulocytes, retaining two decimal places. Inflammation index calculation formula: SII= (neutrophil×platelet)/lymphocyte, SIRI= (neutrophil×monocyte)/lymphocyte, AISI= (neutrophil×monocyte× platelet)/lymphocyte.

Table S3. ROC analysis results for AII, eosinophils, and IgE.

Marker	AUC[a]	95%CI[b]	Sensitivity	Specificity	PPV[c]	NPV[d]	Cut-off[e]
AII	0.568	0.546-0.591	0.614	0.487	0.120	0.910	2.157
Eosinophil	0.546	0.523-0.568	0.361	0.710	0.117	0.901	0.245
IgE	0.542	0.520-0.565	0.711	0.363	0.114	0.908	29.950

a AUC: Area Under the Curve

b 95% CI: 95% Confidence Interval

c PPV: Positive Predictive Value

d NPV: Negative Predictive Value

e Cut-off: Determined based on the threshold that maximizes the Youden's Index (sensitivity + specificity - 1) in the ROC curve analysis.

Table S4. Association between AII and atopic dermatitis, excluded systemic medication history, chronic diseases, tumor history and adult populations respectively.

Variables	Total	Non-atopic dermatitis	Atopic dermatitis	OR (95%CI)[a]	P
**The original results did not rule out medication or underlying diseases**					
Original result	6855	6135(89.50)	720(10.50)	1.03 (1.01 ~ 1.04)	0.003*
**Exclude the history of systemic medication in the past 1 month, n(%)**					
Group 1 (exclude systematic glucocorticoids)	6840	6121(89.49)	719(10.51)	1.03 (1.01 ~ 1.04)	0.003*
Group 2 (exclude antibiotics)	6616	5920(89.48)	696(10.52)	1.03 (1.01 ~ 1.04)	0.002*
Group 3 (exclude NSAIDs[b])	6558	5863(89.40)	695(10.60)	1.03 (1.01 ~ 1.05)	0.002*
Group 4 (exclude immunesuppressants)	6844	6125(89.49)	719(10.51)	1.03 (1.01 ~ 1.04)	0.003*
Group 5 (exclude biologics)	6850	6131(89.50)	719(10.50)	1.03 (1.01 ~ 1.04)	0.004*
Group 6 (exclude all medication above)	6288	5620(89.38)	668(10.62)	1.03 (1.01 ~ 1.05)	0.002*
**Exclude the history of chronic diseases and tumors, n(%)**					
Group 1 (exclude hypertension)	5626	5025(89.32)	601(10.68)	1.03 (1.01 ~ 1.05)	0.009*
Group 2 (exclude diabetes)	6479	5793(89.41)	686(10.59)	1.03 (1.01 ~ 1.05)	0.004*
Group 3 (exclude cardiovascular diseases)	6718	6018(89.58)	700(10.42)	1.03 (1.01 ~ 1.04)	0.003*
Group 4 (exclude liver diseases)	6734	6033(89.59)	701(10.41)	1.03 (1.01 ~ 1.04)	0.002*
Group 5 (exclude kidney diseases)	6755	6047(89.52)	708(10.48)	1.03 (1.01 ~ 1.04)	0.002*
Group 6 (exclude thyroid diseases)	6493	5812(89.51)	681(10.49)	1.03 (1.01 ~ 1.04)	0.002*
Group 7 (exclude tumor diseases)	6572	5898(89.74)	674(10.26)	1.02 (1.01 ~ 1.04)	0.004*
Group 8 (exclude all diseases above)	5129	4852(89.34)	547(10.66)	1.03 (1.01 ~ 1.05)	0.005*
**Exclude the adult populations, n(%)**					
Pediatric populations	2539	2192(86.33)	347(13.67)	1.02 (1.01 ~ 1.05)	0.040*

a: Adjust: gender, race, age, education, PIR, BMI, cotinine, asthma and hay-fever.

b: NSAIDs: Non-Steroidal Anti-Inflammatory Drugs.

OR: Odds Ratio, CI: Confidence Interval, *: P<0.05
